# Bilateral ovarian edema with unilateral ovarian leiomyoma and double inferior vena cava: a case report

**DOI:** 10.1186/s13256-020-02418-5

**Published:** 2020-07-12

**Authors:** Suraj Shrestha, Sushan Homagain, Suraj Kandel, Pooja Jha, Geeta Gurung

**Affiliations:** 1 Maharajgunj Medical Campus, Maharajgunj, Kathmandu, Nepal; 2 Department of Obstetrics and Gynecology, Tribhuvan University Teaching Hospital, Maharajgunj, Kathmandu, Nepal

**Keywords:** Inferior vena cava, Leiomyoma, Massive ovarian edema, Ovary

## Abstract

**Background:**

Ovarian edema, ovarian leiomyoma, and double inferior vena cava are all rare clinical entities. The coexistence of all these entities has not been yet reported in the literature.

**Case presentation:**

We report a case of a 25-year-old nulliparous tamang woman with all these rare clinical entities, who presented with a complaint of right-sided lower abdominal pain. After examination and investigation, an ovarian tumor was suspected and laparotomy was performed during which bilateral ovarian edema with a solid tumor on the left side was identified and left salpingo-oophorectomy was done preserving her right ovary. A histopathological examination confirmed the clinical findings.

**Conclusions:**

As ovarian edema is a rare entity, due to lack of clinical suspicion it is often overdiagnosed as a malignant tumor leading to radical surgery with subsequent loss of hormonal function and early infertility. A high degree of clinical suspicion during the intraoperative period is helpful for diagnosis to avoid unnecessary oophorectomy and infertility.

## Background

Massive ovarian edema (MOE) is a rare benign tumor-like lesion of the ovary, often due to disruption of vascular and lymphatic drainage resulting in the accumulation of fluid within the stroma and subsequent enlargement of the ovary [1]. Ultrasonographic findings are nonspecific and frequently misdiagnosed for malignancy, hence, this results in overtreatment of younger patients with resultant loss of hormonal function and fertility [2]. Here, we report a case of bilateral ovarian edema associated with left ovarian leiomyoma and a double inferior vena cava, which are very rare conditions coexisting in a single patient. Fewer than 200 cases of MOE have been reported to date after it was first described by Kalstone *et al.* in 1969 [2, 3]. Only approximately 70 cases of primary ovarian leiomyoma have been reported in the literature. The coexistence of ovarian edema with ovarian leiomyoma and double inferior vena cava has not yet been reported in the literature.

## Case presentation

A 25-year-old regularly menstruating P_0 + 1_ tamang woman, a 41 homemaker, presented to our center with a complaint of insidious-onset right lower abdominal pain for 1 month. A significant positive history of left leg swelling since birth was present. She had a spontaneous abortion at 4 months of gestation 2 years earlier. She had no history of smoking tobacco or alcohol consumption. Further, there is no family history of any chronic or similar illness.

**Fig. 1:**
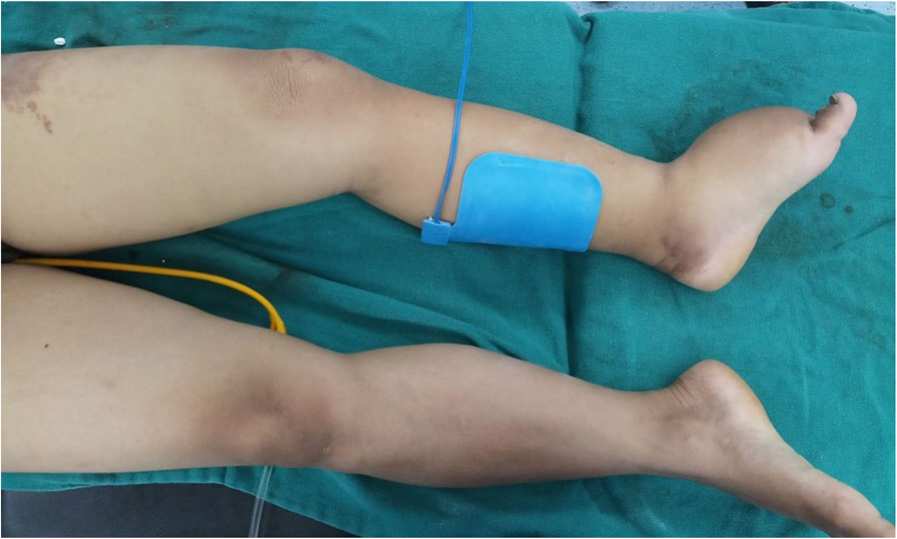
Unilateral left leg swelling extending from foot to upper thigh

At the time of admission, she was afebrile with blood pressure of 110/80 mmHg, regular pulse rate of 88 beats per minute, respiratory rate of 16 breaths per minute, and oxygen saturation of 98% in room air. Her chest was clear on auscultation without any added heart sounds. Neurological examinations were normal throughout. Lymph nodes were not palpable. However, there was non-pitting edema on her left leg extending up to ipsilateral labia majora (Fig. 1). An abdominal examination revealed a 12 cm × 10 cm firm, non-tender, mobile mass in the right side of her lower abdomen. This finding was supported by a bimanual examination. The baseline investigations were within normal limits (Table 1). An abdominal and pelvic ultrasound examination revealed multiple fibroids in her uterus and a large hypoechoic lesion on the right adnexa.

**Table 1:** Baseline investigations

Section	Investigation	Result
**A.**	**Hematological investigations**	
	RBC	4,460,000/μL
	WBC	3700/μL
	DLC	
	a. Neutrophils	78%
	b. Lymphocytes	20%
	c. Eosinophils	1%
	d. Monocytes	1%
	e. Basophils	0%
	Hemoglobin (Hb)	12.8 g/dl
	Platelets	216,000/μL
	Bleeding time	2 minutes (2–7 minutes)
	Clotting time	8 minutes (5–15 minutes)
	Prothrombin time	14 seconds (13–16 seconds)
	INR	1.00
**B.**	**Biochemical investigations**	
	Random blood sugar (RBS)	4.7 mmol/L (3.8–7.8)
	Sodium (Na)	140 mEq/L (135–146)
	Potassium (K)	4 mEq/L (3.5–5.2)
**C.**	**Thyroid function test**	
	Free T4	15.9 pmol/L (10.2–28.2)
	Thyroid-stimulating hormone (TSH)	2.48 microIU/L (0.46–4.68)
	Free T3	4.3 pmol/L (4.26–8.1)
**D.**	**Tumor markers**	
	Carcinoembryonic antigen (CEA)	1.14 ng/ml (< 3 ng/ml)
	Lactate dehydrogenase (LDH)	251 U/L (< 460)
	Alpha-fetoprotein (AFP)	2.16 ng/ml (< 7.5)
	Carbohydrate antigen-125 (CA-125)	21 U/ml (< 35.0)
	Beta-HCG	2.39 μIU/ml (< 5)
	Pap smear	Negative for malignant intra-epithelial lesion
**E.**	**Renal function test**	
	Urea	4.8 mmol/L (1.6–7.0)
	Creatinine	64 mmol/L (40–110)
**F.**	**Serology**	
	1. HIV 1 and 2	Non-reactive
	2. HBsAg	
	3. Anti-HCV	
**G.**	**Urine analysis** (routine and microscopic examination) **Urine culture**	Within normal limits No growth after 72 hours of incubation

*DLC* differential leukocyte count, *HbsAg* hepatitis B surface antigen, *HCG* human chorionic gonadotropin, *HCV* hepatitis C virus, *INR* international normalized ratio, *RBC* red blood cells, *T3* triiodothyronine, *T4* thyroxine, *WBC* white blood cells

**Fig. 2:**
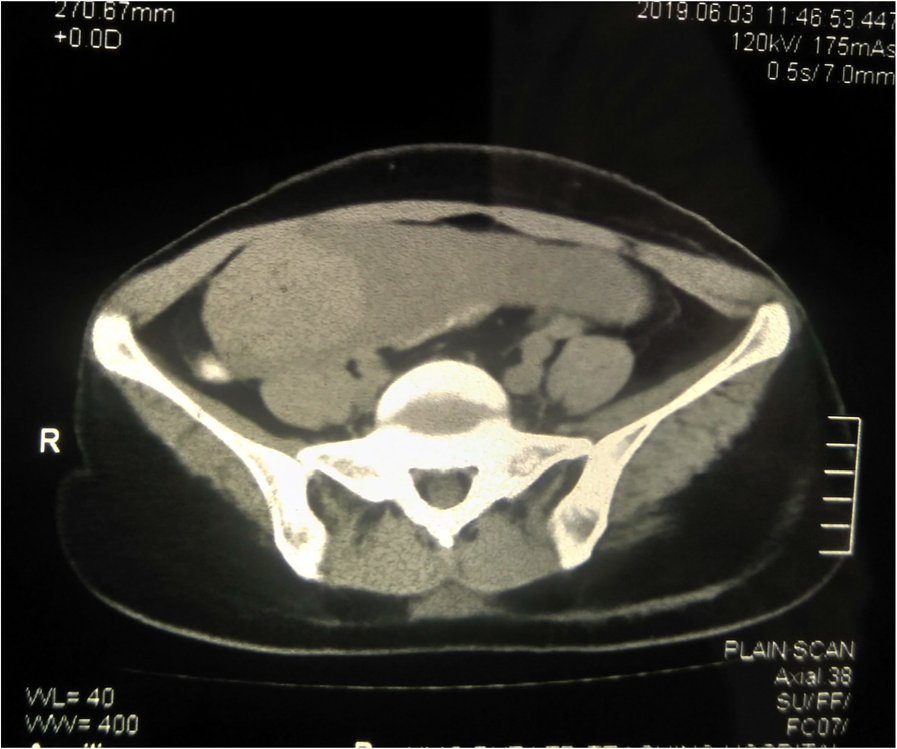
Computed tomography scan of pelvis (axial view) showing solid cystic lesion of left ovary extending to right pelvic region

A computed tomography (CT) scan of her abdomen showed a well-marginated thin-walled solid cystic lesion measuring 16.1 cm × 7.9 cm × 8.1 cm in the abdominopelvic region (Fig. 2). A well-defined cystic lesion measuring 2.3 cm × 2.1 cm and a well-defined solid lesion measuring 5.8 cm × 5.7 cm were noted within the cystic lesion. Another cystic lesion measuring 7 cm × 3.2 cm × 2.8 cm was noted in the right parauterine region with no evidence of calcification and enhancement. Bilateral ovaries were not visualized separately. A CT scan also revealed a lobulated bulky uterus with multiple fibroids, the largest measuring 6.7 cm × 3.4 cm in the anterior wall. Multiple homogenously enhancing mesenteric lymph nodes were noted, the largest measuring 1.3 cm × 1 cm.

**Fig. 3:**
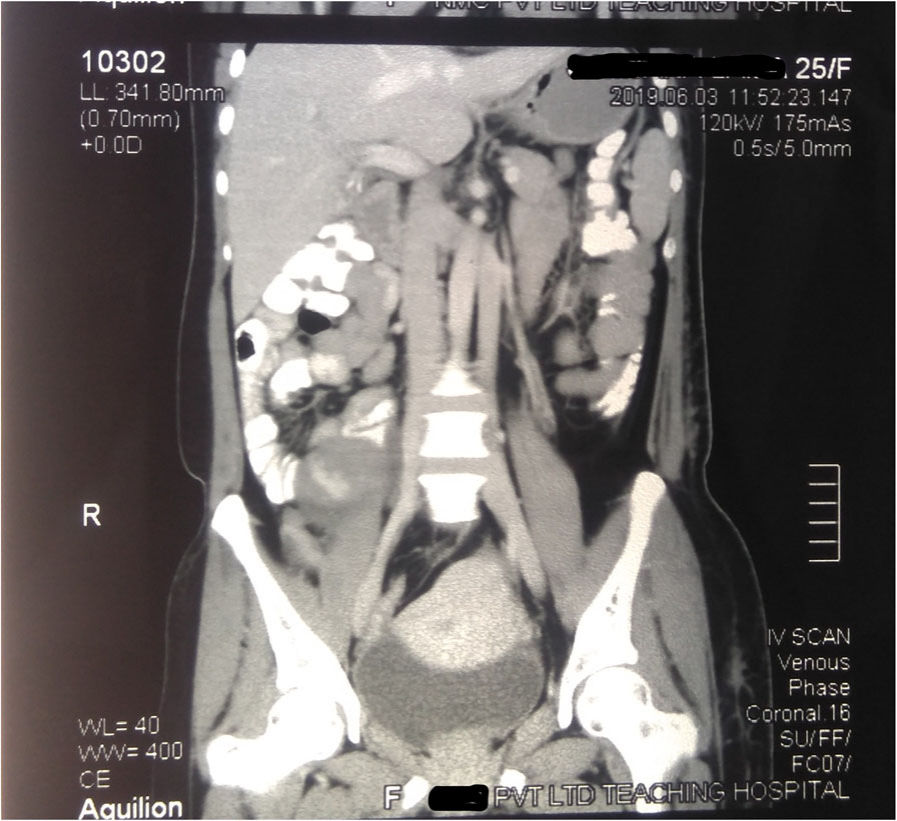
Computed tomography scan of abdomen (coronal view) showing left inferior vena cava as a continuation of left common iliac vein draining into the left renal vein

A rare incidental finding of double inferior vena cava was also noted. The left inferior vena cava was noted as a continuation of left common iliac vein draining into the left renal vein, crossing anterior to the aorta, and joining the right-sided inferior vena cava (Fig. 3). The serology was negative for elephantiasis. Although tumor markers were normal (Table 1), radiological findings were suggestive of an ovarian tumor; a staging laparotomy and surgical excision of the tumor and fibroid was planned with a provisional diagnosis of a malignant ovarian tumor with multiple fibroid and unilateral lymphedema.

**Fig. 4:**
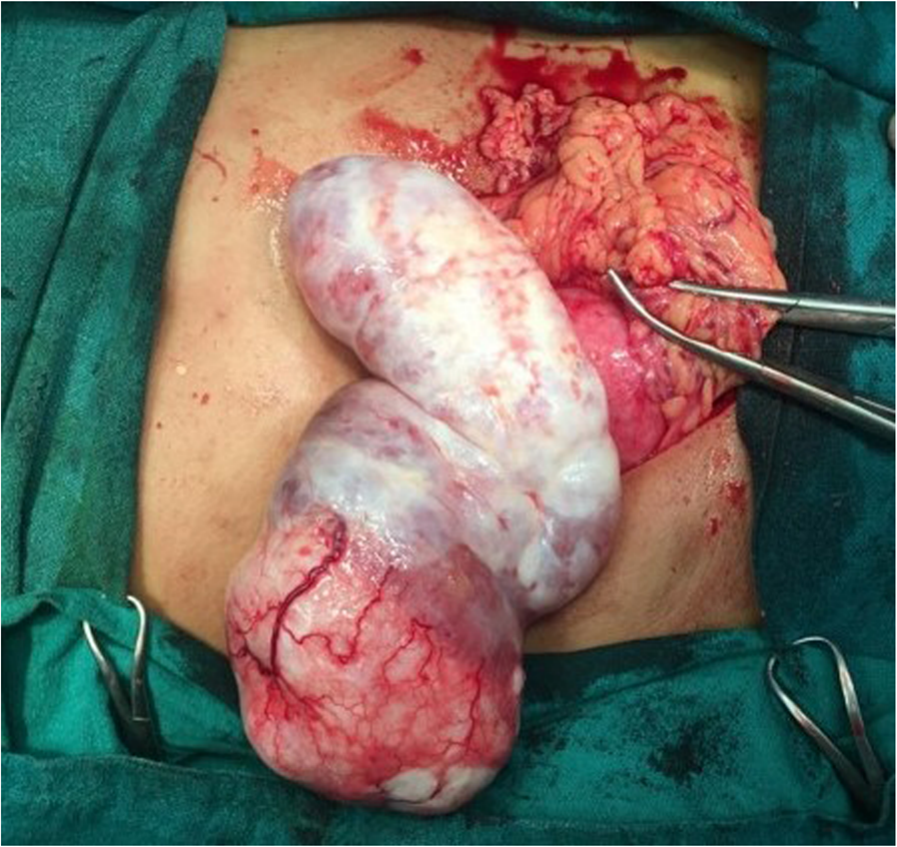
Grossly edematous left ovary with solid component

To our surprise, the left ovary was found to be enlarged measuring around 18 cm × 5 cm with a whitish smooth surface (Fig. 4). The right ovary was also edematous measuring 5 cm × 3 cm in size. There were no signs of torsion, necrosis, and hemorrhage. Bulges were noted in the posterior and superior aspect of the uterus measuring approximately 5 cm × 5 cm and 4 cm × 4 cm.

Hence, conservative surgery was performed as ovaries were edematous bilaterally and the solid lesion on the left showed no signs of invasion and metastasis. A left salpingo-oophorectomy with myomectomy was done. On the cut section, the left ovarian mass showed a solid lesion of 5 cm × 5 cm, and the myomectomy specimen showed multiple lesions with loss of whorled appearance. Her postoperative period was uneventful and she was discharged on the fourth postoperative day with orally administered antibiotics (cefixime 200 mg twice a day for 10 days), analgesics (ibuprofen 400 mg + paracetamol 500 mg three times a day for 3 days then if there was a need along with pantoprazole 40 mg) and multivitamins (vitamin B complex and vitamin C for a month with zinc).

**Fig. 5:**
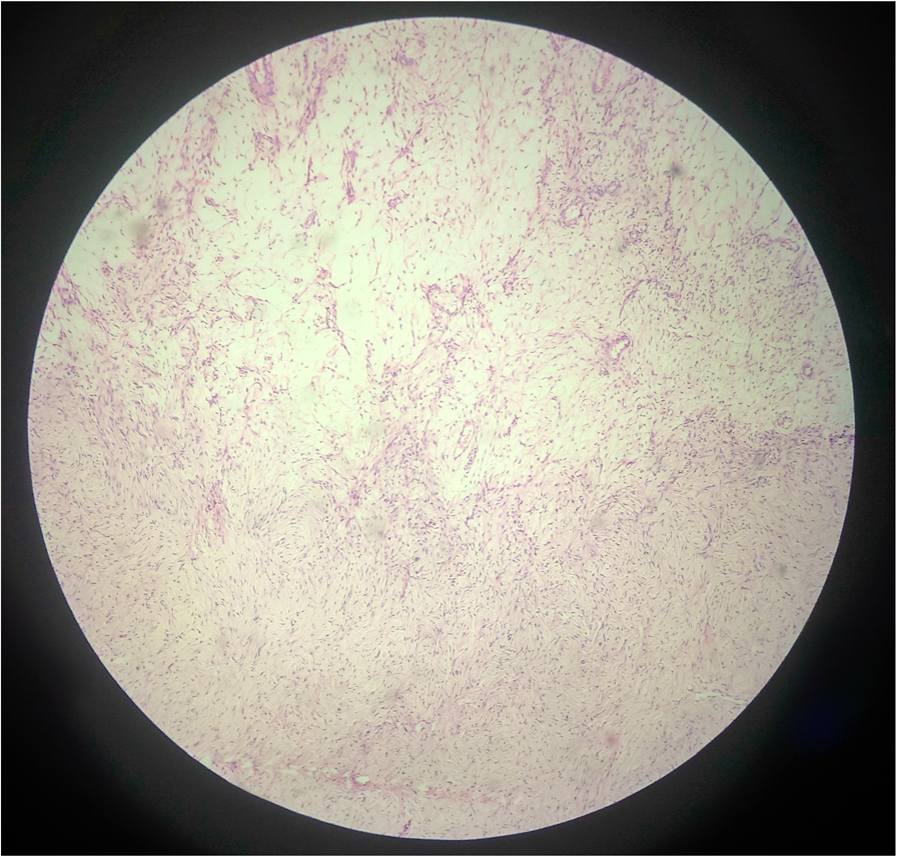
Markedly edematous stroma with scattered spindle cells

A histopathological examination of the left ovary showed features suggestive of ovarian leiomyoma with edema without features of atypia, pleomorphism, mitosis, and necrosis (Fig. 5). Our patient was on regular follow-up. She was doing well and asymptomatic at 9 months of follow-up.

## Discussion and conclusions

Our patient was a 25-year-old nulliparous woman who presented to us with a complaint of right-sided lower abdominal pain. A physical examination suggested a pelvic mass which was confirmed by radiological investigation, which also revealed a double inferior vena cava, a venous anomaly. With a provisional diagnosis of a malignant tumor, staging laparotomy and surgical excision of the tumor was planned. The diagnosis of ovarian edema was made clinically and later confirmed by histopathological examination of the excised mass which also showed ovarian leiomyoma. Our patient had ovarian edema, ovarian leiomyoma, and double inferior vena cava which are rare clinical entities, the coexistence of which has not yet been reported in the literature.

The World Health Organization (WHO) defined ovarian edema as an accumulation of edema fluid within the ovarian stroma separating normal follicular structures [1, 3]. It most commonly affects young women in their reproductive stage, with an average age of 20 years with some cases reported during pregnancy [4]. Of all cases of MOE, 14% develop bilaterally, with right-side predominance [5]. This is attributed to higher pressure in the right ovarian vein compared to the left since it drains directly into the inferior vena cava. As it has been suggested that massive enlargement of the ovary results from interference with the venous and lymphatic flow, the left-sided predominance in our case could be due to the presence of an ovarian leiomyoma on the left side and possible vascular and lymphatic malformation in the pelvic region, as evident in our patient with double inferior vena cava and childhood lymphedema.

Secondary MOE can occur in benign ovarian tumors such as mature cystic teratoma, fibrothecoma, and Meigs syndrome, polycystic ovary, and metastatic malignancies from the uterine cervix, gastric carcinoma, and lymphangitis carcinomatosa, and secondary to the drugs used for ovulation induction [3, 6].

Concurrent pathology such as serous cystadenoma has also been reported with ovarian edema [7]. The presence of ovarian leiomyoma might aggravate ovarian edema as in our case. However, the simultaneous presence of ovarian leiomyoma and ovarian edema has not been described in the literature and might be mere co-incidental findings.

Ovarian leiomyoma is one of the rarest solid tumors of the ovary accounting for only 0.5–1% of all the benign ovarian tumors [8]. Ovarian leiomyomas are particularly unilateral and small, and most commonly occur in women aged 20–65 years. Similar to our case, the majority of these tumors are discovered incidentally, with approximately 80% of the cases occurring in premenopausal women [9].

Despite technological advances, it is difficult to preoperatively diagnose ovarian edema with imaging techniques and the diagnosis is often intraoperative, which requires a high degree of clinical suspicion and experience and can be confirmed on histopathological examination postoperatively which holds for our case. The ultrasound findings are usually reported as a solid tumor, such as a mass or as a solid mass with cystic component, which is nonspecific and can mimic neoplasia [10].

MRI demonstration of multiple ovarian follicles situated around the periphery of the cortex of the enlarged ovary is the most characteristic sign of MOE [5]. MRI can be a very helpful tool in further evaluating the adnexa if ultrasound findings are inconclusive [11].

Radiological imaging in most of the situations can be ambiguous, however, with the addition of tumor markers, the differential diagnosis can be scaled down, differentiating this condition from dysgerminomatous and mixed germ cell tumors [12]. The involved ovary is grossly enlarged, soft, and fluctuant and to diagnose it intraoperatively requires a great deal of clinical experience if the facilities for frozen section are lacking.

On the cut section, thin edema fluid oozes out, and the specimen appears wet and soft. Characteristically, a thin rim of the compressed cortical stroma is recognized at the periphery of the mass with preserved ovarian architecture.

MOE is an unusual cause of ovarian enlargement seen in young patients [10]. Any ovarian neoplasm that may exhibit edematous or myxoid appearance on the cut section can come in the differential diagnosis of ovarian edema, like fibroma, sclerosing stromal tumor, Krukenberg tumor, luteinized thecoma associated with sclerosing peritonitis, and ovarian myxoma. The presence of preserved follicular structures within an edematous stroma can help differentiate the lesion from fibroma and luteinized thecoma [13].

Management of this entity depends on the condition of the patient and may vary from bilateral salpingo-oophorectomy to simple ovarian biopsy [14]. Medical management would not be a practical option as the diagnosis of MOE is usually retrospective and there are no known medical methods available to manage this condition [3].

When an ovarian mass is found in a young woman, frozen section examination can be helpful in diagnosis and to avoid unnecessary oophorectomy and subsequent infertility [1]. The majority of patients are overtreated with salpingo-oophorectomy, as the lesions are mistaken for primary ovarian neoplasm [15]. When the condition of ovarian edema is suspected at surgery, the appropriate treatment is wedge resection, removing 30% or more of the ovary to exclude the secondary causes of the condition [12]. When the diagnosis of MOE is made, every effort should be made to preserve the ovarian function [14].

In our case, ovarian edema was diagnosed clinically during laparotomy. Due to a lack of technological advancement in our institution, the frozen section could not be done. Left salpingo-oophorectomy was performed because there was a solid mass in the left ovary along with ovarian edema. The right ovary was, however, preserved considering the nulliparity and reproductive age group of our patient.

## Conclusion

Most of the cases of ovarian edema are generally overdiagnosed and overtreated due to lack of clinical suspicion and absence of pathognomonic clinical, radiological, or biochemical characteristics. Hence, the knowledge of its existence and the role of the intraoperative frozen section is undoubtedly immense. It should be included in the differential diagnosis in women of reproductive age with enlargement of the ovary.

A high degree of clinical suspicion, experience, and intraoperative frozen section can spare young women from undergoing radical surgery and subsequent loss of hormonal functions and fertility. Furthermore, the presence of unilateral abnormalities adds to the vascular and lymphatic etiology of the pathology.

## Data Availability

All data are within the article.

## Abbreviations

CT: Computed tomography
MOE: Massive ovarian edema
